# Transcriptional down-regulation of the retinoblastoma protein is associated with differentiation and apoptosis in human colorectal epithelial cells

**DOI:** 10.1054/bjoc.2000.1635

**Published:** 2001-02

**Authors:** M Guy, M Moorghen, J A Bond, T J Collard, C Paraskeva, A C Williams

**Affiliations:** CRC Colorectal Tumour Biology Research Group, Department of Pathology and Microbiology, School of Medical Sciences, University of Bristol, University Walk, Bristol, BS8 1TD; Cancer Research Campaign Laboratories, Department of Pathology, University of Wales College of Medicine, Heath Park, Cardiff, CF4 4XN, UK

**Keywords:** retinoblastoma protein, colorectal cancer, differentiation, apoptosis

## Abstract

The aim of this study was to investigate the regulation of Rb protein expression in relation to increased differentiation and induction of apoptosis in colonic epithelial cells. In vivo, Rb protein expression was found to be down-regulated towards the top of the normal colonic crypt, coincident with the region of differentiation and apoptosis, but highly expressed in colonic carcinoma tissue. Using in vitro models to study the regulation of Rb expression in pre-malignant colonic epithelial cells, we have been able to show for the first time that Rb protein expression is transcriptionally down-regulated in differentiated pre-malignant cells (in post-confluent cultures) but not in malignant colorectal epithelial cells. Furthermore, suppression of rb protein function by the HPV-E7 viral oncoprotein increased both spontaneous and DNA damage-induced apoptosis. These results suggest that Rb is able to act as a survival factor in colonic epithelial cells by suppressing apoptosis, and that over-expression of pRb in colorectal tumour cells can cause a loss of sensitivity to apoptotic signalling, resulting in aberrant cell survival and resistance to therapy. © 2001 Cancer Research Campaign http://www.bjcancer.com


*British Journal of Cancer* (2001) **84**(4), 520–528
© 2001 Cancer Research Campaign
doi: 10.1054/ bjoc.2000.1635, available online at http://www.idealibrary.com on IDEAL®

http://www.bjcancer.com

### Transcriptional down-regulation of the retinoblastoma protein is associated with differentiation and apoptosis in human colorectal epithelial cells

**M Guy[1], M Moorghen[1], JA Bond[2], TJ Collard[1], C Paraskeva[1] and AC Williams[1]**

[1]CRC Colorectal Tumour Biology Research Group, Department of Pathology and Microbiology, School of Medical Sciences, University of Bristol, University Walk, Bristol, BS8 1TD; [2]Cancer Research Campaign Laboratories, Department of Pathology, University of Wales College of Medicine, Heath Park, Cardiff, CF4 4XN, UK

**Summary** The aim of this study was to investigate the regulation of Rb protein expression in relation to increased differentiation and induction of apoptosis in colonic epithelial cells. In vivo, Rb protein expression was found to be down-regulated towards the top of the normal colonic crypt, coincident with the region of differentiation and apoptosis, but highly expressed in colonic carcinoma tissue. Using in vitro models to study the regulation of Rb expression in pre-malignant colonic epithelial cells, we have been able to show for the first time that Rb protein expression is transcriptionally down-regulated in differentiated pre-malignant cells (in post-confluent cultures) but not in malignant colorectal epithelial cells. Furthermore, suppression of rb protein function by the HPV-E7 viral oncoprotein increased both spontaneous and DNA damage-induced apoptosis. These results suggest that Rb is able to act as a survival factor in colonic epithelial cells by suppressing apoptosis, and that over-expression of pRb in colorectal tumour cells can cause a loss of sensitivity to apoptotic signalling, resulting in aberrant cell survival and resistance to therapy. © 2001 Cancer Research Campaign http://www.bjcancer.com

**Keywords**: retinoblastoma protein; colorectal cancer; differentiation; apoptosis

The colonic crypt is a constantly self-renewing tissue in which a balance between proliferation, differentiation and cell death must be precisely controlled in order to maintain normal tissue homeostasis. In the normal colon, proliferating cells migrate up the crypt where they differentiate and subsequently undergo what is believed to be p53-independent apoptosis at the lumenal surface (Gaverelli et al, 1992; Hall et al, 1994). This is distinct from the p53-dependent apoptosis that occurs in response to DNA damage, for example, and which is localized to the proliferating stem cell compartment of the crypt (Merrit et al, 1994). The retinoblastoma protein (pRb) has been implicated in cell cycle regulation, differentiation and apoptosis in other tissue systems (Coppola et al, 1990; Haas-Kogan et al, 1995; Sellers and Kaelin, 1996), although its role in the colon and in colorectal carcinogenesis remains unclear.

The Rb protein is encoded by the *RB-1* tumour-suppressor gene, and is a member of the 'pocket protein' family along with two related proteins, p130 and p107. The term 'pocket protein' is derived from the discovery that these 3 proteins have sequence homology in their A/B pocket domain, which is a region of tumorigenic mutations, viral oncoprotein binding and E2F interactions (reviewed in Paggi et al, 1996). Cyclin-cdk complexes regulate the binding of the pocket protein/E2F complexes by phosphorylating the pocket proteins in a cell cycle-dependent manner (reviewed in Beijersbergen and Bernards, 1996). For instance, the interaction of hypophosphorylated pRb with E2F-1 prevents entry into S phase (Weinberg, 1995). Hence pRb and the other members of the pocket protein family perform an important function in the co-ordination of the cell cycle and cell proliferation.

Clues that Rb might play an important role in the differentiation process came initially from studies in knock-out mice. These revealed that *RB*-1 knockout mice die at around day 14.5 of gestation, and exhibit defective differentiation and extensive apoptosis in the haematopoietic system, lens and nervous system (Clarke et al, 1992; Jacks et al, 1992; Lee et al, 1992; Zackenhaus et al, 1996), suggesting that Rb is required for the differentiation of specific tissues. Additionally, in vitro experiments have shown that hypophosphorylation of the Rb protein may play a role in the differentiation of some cell types, e.g. human haematopoietic cells (Furukawa et al, 1990), leukaemia cell lines (Chen et al, 1989), PC12 neuronal cells (Kalman et al, 1993; Li et al, 1996) and myoblast cell lines (Gu et al, 1993). Interestingly, while differentiation in erythroleukaemia cells (Coppola et al, 1990; Richon et al, 1992), myocytes (Coppola et al, 1990; Endo and Goto, 1992), and embryonal carcinoma cells (Slack et al, 1993) is associated with an accumulation of *RB-1* mRNA, the other pocket proteins do not appear to play such a crucial role in differentiation. Indeed, mice deficient for either p107 or p130 show no developmental defects (Cobrinik et al, 1996), indicating that the pocket protein family may functionally compensate for one another.

Previous studies suggest an involvement of pRb in the regulation of apoptotic cell death (reviewed in Dou and An, 1998). Experiments using the human osteosarcoma cell line SAOS-2, which lacks functional pRb, showed that radiation-induced apoptotic cell death was high in the absence of Rb protein in comparison to SAOS-2 cells transfected with wild-type pRb (Haas-Kogan et al, 1995). Similarly Almasan et al (1995) reported that the

*Received 22 June 2000*
*Revised 2 November 2000*
*Accepted 8 November 2000*

*Correspondence to:* M Guy targeted inactivation of the *RB-1* gene in mouse embryonic fibroblasts induces apoptosis. Therefore, pRb appears to suppress apoptosis in some cell systems, perhaps through the sequestration of the E2F-1 protein, which can induce apoptosis if over-expressed (Qin et al, 1994; Field et al, 1996). In this case, pRb needs to be removed or inactivated before apoptosis can occur. To support this, a 30 kD protein has been detected in apoptotic cells harvested from colon tumour cell lines, CMSV40 fibroblasts and BJA-B lymphocyte cells, suggesting that the Rb protein is cleaved during the apoptotic process (Browne et al, 1994; An and Dou, 1996). Furthermore, cleavage at the C-terminus of pRb results in a p100 and ~5 kDa polypeptide (Chen et al, 1997). The p100 Rb protein can still bind to E2F-1 and inhibit E2F-mediated transcriptional activity, but its anti-apoptotic function is reduced, perhaps through its inability to bind to the oncogene MDM2. Thus the growth-suppressive and anti-apoptotic functions of pRb appear to be distinct from one another. The notion that pRb functions can be separated from one another has been explored previously, and experimental evidence indicates that the multiple functions of pRb can be genetically uncoupled, providing distinct functions in cell-cycle control or in tissue-specific gene expression during differentiation (reviewed in Yee et al, 1998). Mice with mutations of the N-terminal region of Rb protein exhibit defects in muscle differentiation, whilst retaining the ability to bind to E2F (Riley et al, 1997). Conversely, cells with pRb mutations in the pocket domain are able to differentiate normally even when E2F binding is abrogated (Sellers et al, 1998).

The functional loss of the Rb protein, by deletion or mutation, has been implicated not only in retinoblastoma, but in many types of tumour, including bladder, breast, lung and ovarian cancer (Berns et al, 1995; Hiyana et al, 1995; Takano et al, 1995; Miyamoto et al, 1996). However, loss or inactivation of the *RB-1* gene in colorectal tumours is uncommon (Meling et al, 1991; Ali et al, 1993); only up to 11% of colorectal carcinomas show allelic loss on chromosome 13 (Wildrick and Bowman, 1994). Instead, colorectal tumours have been reported to express significantly higher levels of *RB-1* mRNA than normal colonic mucosa (Gope et al, 1990). This increase coincides with a 1.5–2.5 fold increase in percentage of pRb phosphorylation (Gope and Gope, 1992), which is thought to be due to over-expression of the Rb-related kinases cdk2 and cdc2 (Yamamoto et al, 1995). In addition, Rb protein associated with elevated levels of transcripts appears to be normal size (4.7 kb) and functional (Ali et al, 1993), suggesting that colorectal cancer cells retain functional pRb.

There are few previous studies on the role of the Rb pocket protein in differentiation and apoptosis of colonic epithelial cells. One in vivo study reports that Rb protein staining was localized preferentially to the midcrypt differentiation zone in normal human colonic epithelium; while fully differentiated cells at the top of the crypt were pRb negative (Ali et al, 1993). However, the authors' state that these results were not conclusive, as the possibility of cross-reaction of the pRb antibody with other members of the pocket protein family could not be ruled out. Therefore the aim of this study was to investigate the regulation of expression of Rb protein in relation to increased differentiation and induction of apoptosis in colonic epithelial cells. Initially we studied the differential protein expression of Rb and the related pocket proteins (p107 and p130) in the normal colonic crypt in vivo. Results showed that down-regulation of the Rb protein correlated with the region of functional differentiation and apoptosis in the normal colonic crypt, and that Rb protein was highly expressed in colorectal carcinomas. Therefore, we used an in vitro model system to investigate the mechanism of regulation of pRb expression with increased differentiation in premalignant colorectal epithelial cells (by maintenance at confluence or treatment with the differentiation agent sodium butyrate). In addition we investigated whether suppression of pRb function increased the susceptibility of pre-malignant adenoma-derived cells to apoptosis.

### MATERIALS AND METHODS

### Immunostaining of normal human colonic crypts and tumour tissue

Formalin-fixed paraffin embedded tissues were used in this study. These were retrieved from the archives of the Department of Histopathology, Bristol Royal Infirmary. 10 samples of colorectal adenocarcinomas (taken from colectomy specimens from 10 different patients) at various grades of differentiation were examined. In 4 of these cases adjacent mucosa which appeared morphologically normal in H&E sections were also examined. A further 6 samples of normal mucosa were obtained from areas situated at least 6 cm from the tumour mass in colectomy specimens. pRb C-15 Antibody (Pharmingen) was used at a dilution of 1:2000, p130 (C-20) and p107 (C18) antibodies (Santa Cruz) were diluted 1:500. A Biotin Streptavidin amplified detection system was used. For antigen retrieval the pRb and p130 sections were microwaved at medium power for 20 minutes in citrate buffer, while the p107 sections were pressure cooked for 100 seconds in EDTA buffer. For each antibody preliminary experiments were carried out to establish optimal dilutions and antigen-retrieving protocol. For the pRb antibody, positive staining of lymphocytes in the tissue examined provided a useful internal control. In a number of cases there was poor and inconsistent staining of background lymphocytes. These were excluded from the study. Blocking peptides for each antibody (Pharmingen and Santa Cruz) were used to verify that there was no cross-reactivity of antibodies (data not shown). Slides were assessed by an independent pathologist.

### Cell culture

Human colorectal adenoma and carcinoma-derived cell lines were used in this study. PC/AA/C1 is a clonogenic, non-tumorigenic adenoma cell line (Paraskeva et al, 1984), which has been converted in vitro to a tumorigenic derivative cell line (tumorigenic in nude mice) designated PC/AA/C1/SB10 (Williams et al, 1990). These two cell lines consequently represent a relatively early and late stage in the multi-step process of colorectal carcinogenesis. Both cell lines were grown in conditioned medium (described in Williams et al, 1990). S/RG/C2 is a clonogenic cell derived from a sporadic tubular adenoma and grown in 20% FBS DMEM (Life Technologies, UK) (Paraskeva et al, 1984). PC/JW2 is a carcinoma-derived cell line (Paraskeva et al, 1984) which was maintained in 10% FBS DMEM.

### Infection of E7 cell lines

The PC/AA/C1 cell line was infected with the HPV E7 oncoprotein using retroviral gene transfer (as described in Bond et al (1999) for thyroid epithelial cells) and maintained in G418 selection medium at a concentration of 200 $\mu g\ ml^{-1}$. The resultant cell line was designated AA/C1/RE7, the corresponding vector control

© 2001 Cancer Research Campaign

AA/C1/NEO. E7 expression was verified by immunostaining with HPV-16 E7 monoclonal antibody (Ciba Corning) (Jane Bond, data not shown). It is important to note that both the HPV E7-infected cell line (AA/C1/RE7) and vector control cell line (AA/C1/NEO) were isolated from several hundred pooled colonies which had survived the drug selection.

### Induction of differentiation using cell confluency

Differentiation was induced by allowing the cells to grow to post-confluency. Once confluent cells become non-cycling they begin to differentiate as indicated by cell morphology. Triplicate flasks of cells were grown to 50% confluency, 100% confluency, one week post-confluent and two weeks post-confluent. At these time-points the cell monolayers were trypsinized to single cells, and samples prepared for Western blotting by the method described in Williams et al (1999).

### Induction of differentiation using sodium butyrate

Cells were seeded at a density of $2 \times 10^6$ per flask in triplicate flasks and left to grow exponentially for 72 hours. A stock solution of 100 mM sodium butyrate (Sigma, UK) was prepared in tissue culture water and diluted in the appropriate medium to concentrations of 1 to 3 mM (representing normal physiological concentrations; Cummings, 1981). Cells were treated for 48 hours before trypsinization to single cells. Control cultures were treated with growth medium and tissue culture water only.

### Protein expression by SDS-PAGE

Western samples of $1 \times 10^6$ cells were prepared by the method described in Williams et al (1999). Proteins were resolved on 7.5% polyacrylamide gels and transferred onto Immobilon-P polyvinylidene difluoride membranes (Millipore, MA). Rb protein was detected by the monoclonal antibody Rb245 (Pharmingen), (which detects both hypophosphorylated and hyperphosphorylated forms of pRb), at a 1:1000 dilution and a horse-radish peroxidase-conjugated goat anti-mouse secondary antibody (1:1000 dilution) (Sigma, UK). This secondary antibody was also used for monoclonal anti-α-tubulin (Sigma, UK) which was diluted 1:10 000 to control for loading. p107 and p130 proteins were detected using C-18 (1:200 dilution) and C-20 (1:500 dilution) polyclonal antibodies respectively (Santa Cruz) and horse-radish peroxidase-conjugated goat anti-rabbit secondary antibody (1:1000 dilution) (Sigma, UK). Protein bands were visualized using the Amersham enhanced chemiluminescence detection system following the manufacturer's protocol.

### Northern blotting

$1 \times 10^7$ cells were washed in PBS and total RNA was extracted using the Qiagen (Chatsworth, CA) RNEasy minikit according to the instructions of the manufacturer. 13 μg of total RNA was separated on a 0.9% agarose gel, containing 3% formaldehyde, and transferred to a nylon membrane (Genescreen Plus, NEN Life Sciences, MA). α-$^{32}$P-dATP labelled probes for *RB-1* (template wtpRb cDNA was from Sybille Mittnacht, ICR, London) and 18S loading control (template from Maria Davies, Dental School, University of Bristol) were prepared using a random-primer DNA-labelling kit (Stratagene, CA) and hybridized overnight at 68˚C. Filters were washed to a final stringency of 0.2 × SSC/1% SDS (10 min, 68˚C) and bands visualized by autoradiography.

### Assessment of apoptosis

The level of apoptosis was measured by the method described in Williams et al (2000). Briefly, the level of apoptosis in cultured epithelial cell lines was assessed by measuring the proportion of cells that detached from the monolayer and were floating in the medium, and by determining the fraction of these floating cells that were apoptotic. The attached and floating cell populations were stained with 5 μg ml$^{-1}$ acridine orange in PBS, and analysed by fluorescent microscopy for morphological features of apoptosis (most obviously the characteristically condensed chromatin). Analysis was carried out by an experienced observer unaware of the cell type or treatment. The fraction of floating cells that were apoptotic did not significantly vary between treated and control untreated cell populations and therefore the number of floating cells could be used as a measure of the induction of apoptosis.

### RESULTS

### Rb protein and related proteins p107 and p130 are differentially expressed in the normal human colonic crypt in vivo

As differential expression of proteins along the axis of the normal colonic crypt has previously given important clues as to their function in the colonic crypt (for example Avery et al, 1993; Williams et al, 2000), the expression of pRb and related pocket proteins (p107 and p130) was investigated for at least 10 sections from different patients of normal colonic mucosa and tissue adjacent to colon tumours where appropriate. Blocking peptides for each antibody were used as a control to ensure that antibody staining was specific (refer to materials and methods, data not shown). Results indicate that there is differential expression of all 3 pocket proteins along the length of the normal human colonic crypt. p107 expression was confined to the base of the crypt, in the proliferation zone (Figure 1B), and p130 expression was restricted to the top of the crypts, coincident with the region of differentiation and apoptosis (Figure 1C). These results are consistent with the putative roles of the pocket proteins, as p107 is thought to play a role in cell proliferation, and p130 is associated with maintenance of a differentiated state (reviewed in Mulligan and Jacks, 1998). However, the localization of the Rb protein was quite different from that reported in other tissue types. Although the pRb antibody, which detects both phosphorylated and unphosphorylated forms of the protein, stained throughout the base and mid-crypt, expression was found to be decreased towards the top of the crypt, coincident with the region of differentiation and apoptosis (Figure 1A). Of further interest, expression of the Rb protein was investigated in colorectal adenocarcinomas (results obtained from a minimum of 10 sections from separate patients). In all tumours studied, Rb protein expression was detectable with all cells staining strongly positively for pRb (Figure 1D), suggesting that expression of the Rb protein is selected for in tumour progression.

 © 2001 Cancer Research Campaign

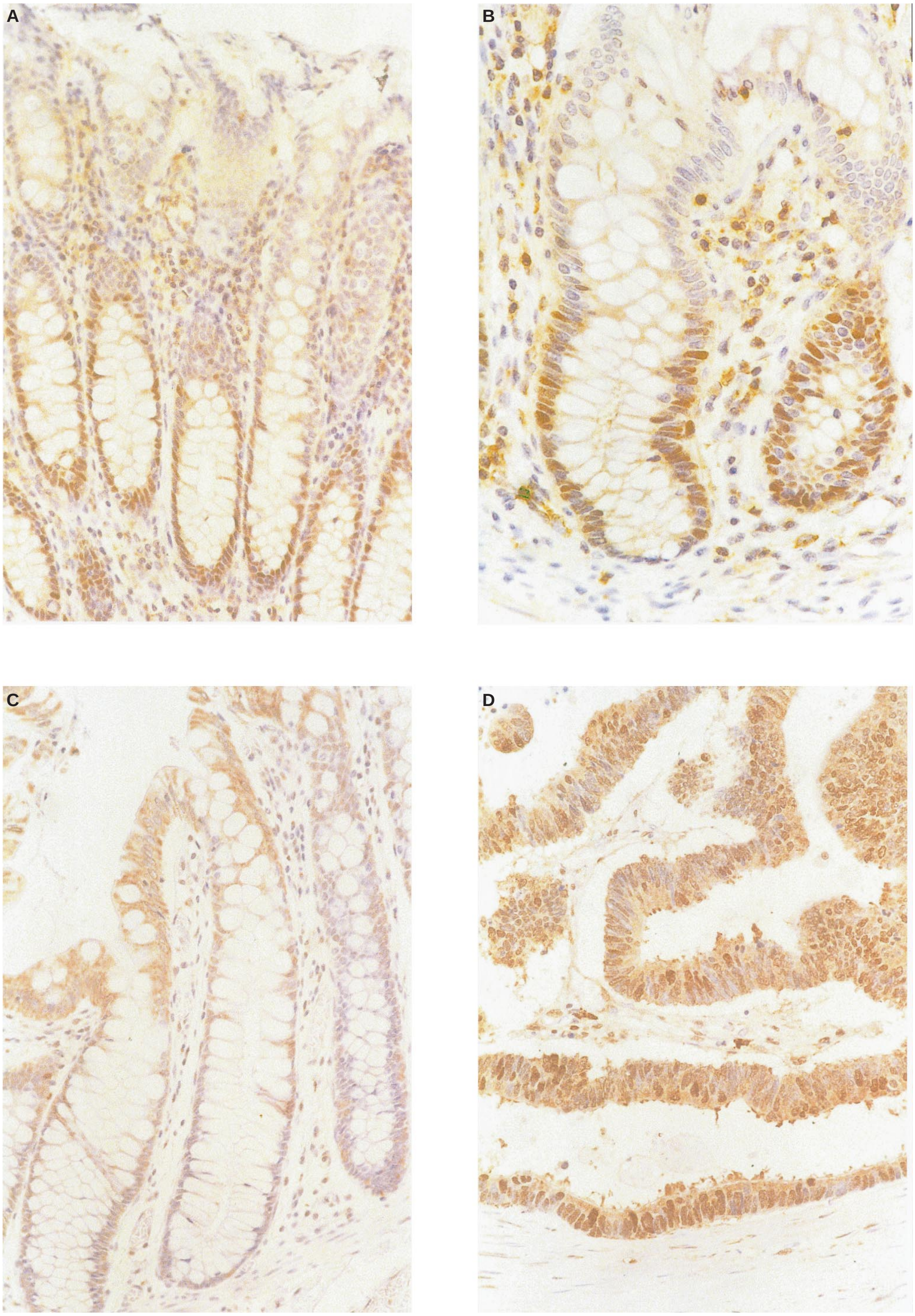

**Figure 1** Immunostaining of normal human colonic epithelium tissue sections. (**A**) Rb protein expression in normal colonic epithelium using the Rb C-15 antibody (Pharmingen). Although clearly detected throughout the base and mid-crypt, pRb expression is decreased towards the lumenal surface of the colonic crypt. (This antibody does not distinguish between hypophosphorylated and hyperphosphorylated forms of pRb). (**B**) p107 expression in normal colonic epithelium using the C-18 antibody (Santa Cruz). p107 expression is detected at the base of the crypts coincident with the region of proliferation. (**C**) p130 expression in normal colonic epithelium using the C-20 antibody (Santa Cruz). The p130 protein is expressed towards the top of the crypt co-incident with the region of differentiation and apoptosis. (**D**) An adenocarcinoma stained with the Rb C-15 antibody (Pharmingen), showing over-expression of pRb. Results are representative of specimens taken from 10 different patients. Negative controls (blocking peptides) were used as described in the Materials and methods, to ensure that antibody binding was specific

*© 2001 Cancer Research Campaign*

### An increase in differentiation status using cell confluency results in a substantial decrease in Rb protein expression in the PC/AA/C1 adenoma-derived cell line, but not in the tumorigenic derivative PC/AA/C1SB10

Having established that Rb protein expression is decreased towards the top of the crypt, we used an in vitro cell culture model to study the mechanism of regulation of pRb expression with increased colonic epithelial cell differentiation, as this has not been previously reported. The cell lines used were the non-tumorigenic adenoma-derived PC/AA/C1 cell line and a tumorigenic derivative of the PC/AA/C1 cell line, designated PC/AA/C1/SB10. These cell lines represent a well characterized in vitro model of the adenoma to carcinoma sequence (Williams et al, 1990). FACs analysis was used to show that all cell lines enter a $G_1$ arrest when confluent; there was between a 1.4 and 3.6 fold increase in the $G_1$/S ratio when cells become confluent, depending on the cell line (data not shown). Cells were then left for a further 2 weeks, at which stage the cell cultures show clear morphological changes (doming) associated with functional differentiation of the epithelial monolayer (referred to as post-confluent). ‘Domes’ appear as the cells differentiate into columnar epithelial cells, which transport fluid through their lumenal surface and out through their basal surface (Kirkland, 1985). Both the adenoma-derived PC/AA/C1 and the tumorigenic PC/AA/C1/SB10 cell lines form domes in post-confluent cultures (an example of a PC/AA/C1 doming culture is shown in Figure 2B). Western blot analysis of PC/AA/C1 showed that sub-confluent cells express high levels of Rb protein, which is present in both the hyper-and hypo-phosphorylated forms (Figure 3A). On reaching confluency there was a shift from the hyper-phosphorylated to the hypo-phosphorylated form (Figure 3A), coincident with induction of $G_1$ arrest. Interestingly, this was followed by a significant down-regulation of Rb protein expression in 2-week post-confluent cultures (Figure 3A). Furthermore, in a second adenoma-derived cell line, S/RG/C2, a similar down-regulation of Rb protein expression was seen when differentiation was increased in post-confluent cultures (results not shown). In addition, although doming was detected in the post-confluent cultures of the tumorigenic derivative PC/AA/C1/SB10, the down-regulation of Rb protein expression was notably less in this cell line (Figure 3B) and in the carcinoma-derived cell line PC/JW2 (data not shown), suggesting that regulation of Rb protein expression may be aberrant in the tumorigenic cells.

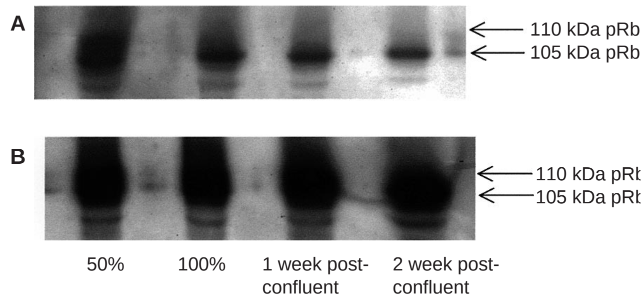

**Figure 3** The effect of increased confluency on pRb protein expression in the human colorectal adenoma cell line (PC/AA/C1) and its tumorigenic derivative (PC/AA/C1/SB10). (**A**) Western blot showing Rb protein expression in the PC/AA/C1 adenoma cell line as the confluency of the cell cultures is increased from 50% confluent to 2 weeks post-confluent. An increase in cell confluency resulted in a switch to the hypophosphorylated form followed by a down-regulation of Rb protein. The antibody used (Rb245, Pharmingen) detects both hyperphosphorylated (110 kDa) and hypophosphorylated (105 kDa) forms of the protein. (Similar results were obtained for the S/RG/C2 adenoma derived cell line, data not shown.) (**B**) Western blot of Rb protein expression in the PC/AA/C1/SB10 tumorigenic derivative when differentiation status of the cells is increased by allowing the cells to become post-confluent. PC/AA/C1/SB10 cells show no down-regulation in Rb protein expression, suggesting that pRb is deregulated during tumour progression. (Similar results were obtained for the PC/JW2 carcinoma derived cell line, data not shown.) Blots were reprobed with anti-α-tubulin as a control for equal lane loading. The results are typical of at least 3 independent experiments, where pRb was reproducibly down-regulated during confluency in PC/AA/C1 but not in the transformed variant PC/AA/C1/SB10

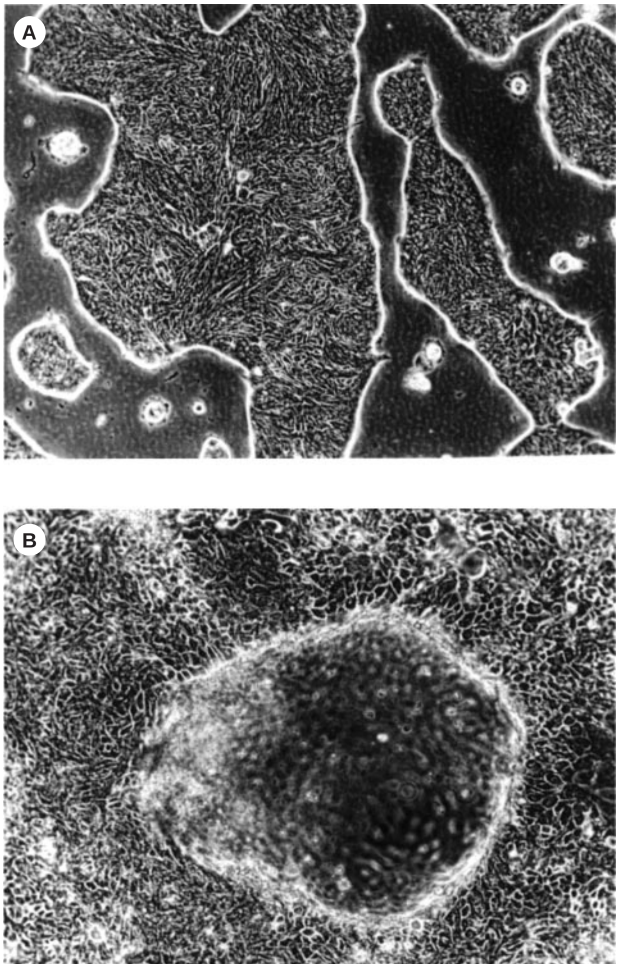

**Figure 2** Morphology of PC/AA/C1 cell line at different stages of confluency. (**A**) Sub-confluent PC/AA/C1 cells. (**B**) Two-week post-confluent PC/AA/C1 cells showing doming of the cell monolayer. This morphological change is seen when the cells are actively transporting fluid and indicates that the cells have become differentiated and functional (Kirkland 1985)

### Treatment with the differentiation agent sodium butyrate results in a decrease in Rb protein expression in the adenoma cell line PC/AA/C1 but not the tumorigenic derivative PC/AA/C1/SB10

To further investigate Rb protein expression in differentiated colonic epithelial cells, we examined the expression of pRb in a second in vitro cell culture model of colonic epithelial cell differentiation. The short-chain fatty acid sodium butyrate (NaBt), a natural fermentation product of dietary fibre (Cummings, 1981)

 *© 2001 Cancer Research Campaign*

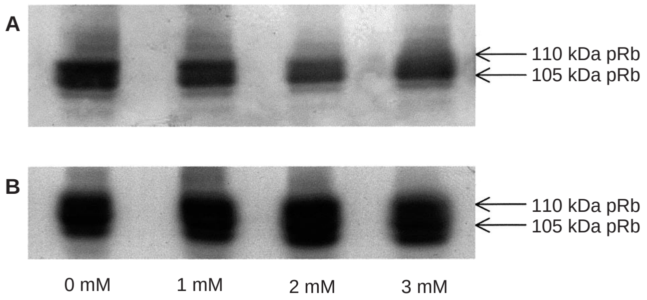

**Figure 4** Effect of sodium butyrate on Rb protein expression in the human colonic adenoma cell line, PC/AA/C1 and the tumorigenic derivative cell line PC/AA/C1/SB10. Western blots showing Rb protein expression (using the Rb245 antibody, Pharmingen) after 48 hour treatment with the differentiation agent sodium butyrate, at concentrations of up to 3 mM. (**A**) In the adenoma cell line, PC/AA/C1, Rb protein expression decreased in a dose-dependent manner. (Similar results were obtained for the S/RG/C2 adenoma derived cell line, data not shown.) (**B**) The tumorigenic derivative cell line PC/AA/C1/SB10 showed no decrease in expression of Rb protein. (Similar results were obtained for the PC/JW2 carcinoma derived cell line, data not shown.) Blots were reprobed with anti-α-tubulin as a control for equal lane loading. The results are typical of at least 3 independent experiments

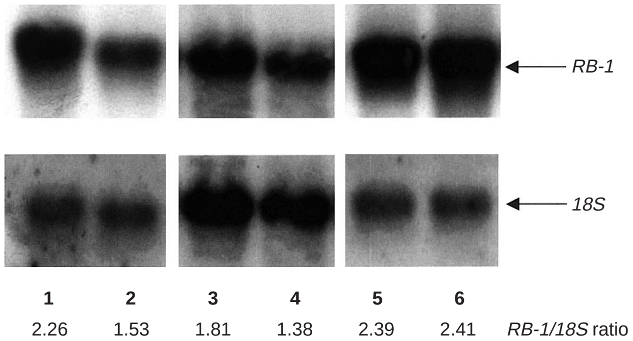

**Figure 5** Effect of increasing confluency on *RB-1* mRNA expression in colonic adenoma and carcinoma cell lines. Expression of *RB-1* mRNA in 50% confluent cells and two week post-confluent cells. Lane 1, S/RG/C2 50% confluent cells; lane 2, S/RG/C2 2-week post-confluent cells; lane 3, PC/AA/C1 50% confluent cells; lane 4, PC/AA/C1 2-week post-confluent cells, lane 5, PC/AA/C1/SB10 50% confluent cells; lane 6, PC/AA/C1/SB10 2-week post-confluent cells. 18S was used to control for RNA loading. The ratio of *RB-1*:18s RNA is shown under the appropriate lanes, as assessed by densitometry. Expression is down-regulated in the S/RG/C2 (33%) and to a lesser extent in the PC/AA/C1 adenoma cell line (24%), but not in the transformed adenoma, PC/AA/C1/SB10. Data shown are representative of results from duplicate experiments has been found to induce differentiation in a number of different cell types including colonic cells (Leder and Leder, 1975; Prasad and Sinha, 1976). Furthermore, it has previously been established that NaBt can induce differentiation in colorectal cell lines, and this induction of differentiation is evidenced by increased expression of the differentiation markers alkaline phosphatase (ALP) and E-cadherin (Butt et al, 1997). On treatment with NaBt, an analogous level of induction of both differentiation markers (ALP and E-cadherin) was detected in both adenoma- and carcinoma-derived cell lines, indicating that an increase in differentiation was detectable in all cell lines (the tumorigenic cell lines PC/AA/C1/SB10 and PC/JW2 as well as the non-tumorigenic PC/AA/C1, S/RG/C2 cells) (Butt et al, 1997). Additionally it has been established using FACs analysis that the $G_1$/S ratio increases in a dose-dependent manner in the colorectal cell lines used in our study after 48 hours of NaBt treatment (approximately 1.5–3.2 fold, depending on cell line; Butt, 1996). Therefore the PC/AA/C1 adenoma cells and their tumorigenic derivative PC/AA/C1/SB10 cells were treated with doses of sodium butyrate (0–3 mM for 48 hours) which had previously been shown to induce differentiation, and the Rb protein expression determined by Western analysis. Figure 4A shows the down-regulation of Rb protein expression with butyrate treatment in a dose-dependent manner in the cell line PC/AA/C1. Similar results were found for the S/RG/C2 adenoma cell line (data not shown). However, the tumorigenic derivative cell line, PC/AA/C1/SB10, showed little or no down-regulation of Rb protein when treated with butyrate (Figure 4B); similar results were obtained for the tumour-derived cell line PC/JW2 (data not shown). Again these results suggest that deregulation of pRb expression may occur in the tumorigenic cells as Rb protein expression is markedly down-regulated in pre-malignant adenoma-derived cells but not in the tumorigenic cells, even though the differentiation status of the cells is similarly increased by NaBt treatment.

### The down-regulation of Rb protein expression is regulated at the transcriptional level

Having established that an increase in the differentiation status of cells resulted in a down-regulation of Rb protein expression in the adenoma-derived cell lines, we wished to determine whether down-regulation of pRb was regulated at the transcriptional level, or by post-transcriptional modification of the Rb protein. Total RNA was extracted from sub-confluent (50% confluent) cells and 2 weeks post-confluent cultures, and *RB-1* mRNA expression was measured using Northern blotting analysis. Figure 5 shows a decrease in *RB-1* mRNA in the two adenoma cell lines; *RB-1* mRNA is significantly decreased in the post-confluent S/RG/C2 adenoma derived cells (33%) and to a lesser extent in the in the 2 weeks post-confluent PC/AA/C1 cell line (24%). However, by contrast, *RB-1* mRNA expression fails to down-regulate in the 2 week post-confluent cells of the tumorigenic derivative adenoma cell line PC/AA/C1/SB10 (Figure 5). This is consistent with the failure of this cell line to down-regulate Rb protein expression as the differentiation status of the cells is increased (Figures 3 and 4). This is the first report that down-regulation of Rb protein expression in adenoma-derived cell lines with increased differentiation is regulated via a transcriptional mechanism, and this regulation of expression is aberrant in the tumorigenic cell line.

### Expression of the HPV E7 oncoprotein in the PC/AA/C1 cell line leads to increased spontaneous apoptosis and an increased apoptotic response after DNA damage

As down-regulation of pRb occurs at the top of the colonic crypt, we wished to determine whether suppression of pRb function in the PC/AA/C1 adenoma cell line was able to affect either the spontaneous rate of apoptosis and/or the cellular response to DNA damage. To suppress pRb function the HPV E7 oncoprotein (which binds to and inactivates pRb) was stably expressed in the PC/AA/C1 cell line by retroviral infection, and expression was verified by immunostaining with an E7 antibody (refer to methods, data not shown). The resultant E7 cell line, AA/C1/RE7, and vector control line, AA/C1/NEO, were both isolated from at least several hundred colonies all expressing the appropriate E7 or vector control. The PC/AA/C1 cell line was selected for these experiments as it has a low endogenous spontaneous rate of apoptosis, and is relatively insensitive to DNA damage-induced

© 2001 Cancer Research Campaign

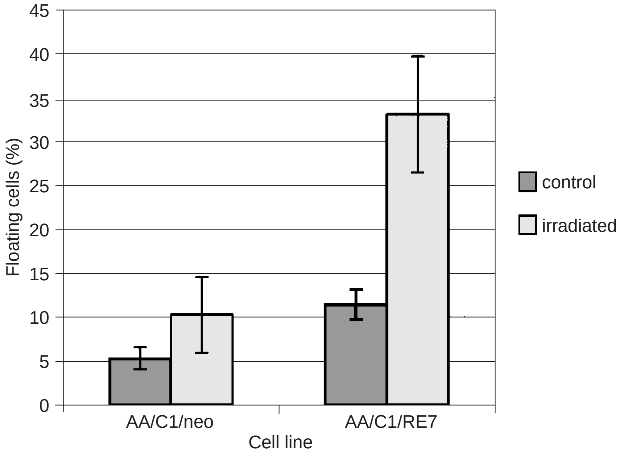

**Figure 6** Effect of suppressing pRb function on apoptosis, by stably expressing the HPV E7 oncoprotein in the AA/C1 cell line. Graph shows radiation-induced apoptosis in E7-infected PC/AA/C1 cells, AA/C1/RE7, 72 hours after exposure to 5 Gy γ-radiation, compared to their vector control, AA/C1/NEO. Percentage of floating cells is used as a measurement of apoptosis (refer to Methods). The results shown represent the mean of 3 independent experiments ± S.D

apoptosis after exposure to 5 Gy γ radiation (Bracey et al, 1997). The level of spontaneous apoptosis was determined in both the E7 expressing cell line (designated AA/C1/RE7) and vector control cell line (designated AA/C1/NEO) by assessing the attached and floating cell yields from exponentially growing cultures after 72 hours. As we and others have described previously, the level of apoptosis in cultured epithelial cell lines can be assessed by measuring the proportion of cells that have detached from the monolayer and are floating in the medium and by determining the fraction of these floating cells that are apoptotic using acridine orange staining to confirm morphological features of apoptosis (Hague et al, 1993; Tsujii and Dubois 1995; refer to materials and methods section). A 1.5-fold increase was observed in spontaneous apoptosis in the AA/C1/RE7 cell line when compared to the vector control cell line (data not shown). In addition, cells (AA/C1/RE7 and AA/C1/NEO) were irradiated with 5 Gy γ radiation, and attached and floating cell yields determined after 72 hours. Results shown in Figure 6 show an increase in γ-radiation-induced apoptosis when Rb protein function is compromised by the expression of the E7 protein, (mean of 3 independent experiments). These results suggest that suppression of pRb function does increase both the rate of spontaneous apoptosis and the level of irradiation-induced apoptosis in the PC/AA/C1 cell line.

### DISCUSSION

The Rb protein is known to play an important role in the cell cycle regulation, differentiation and apoptotic pathways in many different cell systems. As these processes are critical in the maintenance of tissue homeostasis in the colonic crypt, it is somewhat surprising that relatively little is known about the specific role of the Rb protein in the colon. The differential expression of other proteins within the colonic crypt, for example Bcl-2 (Hague et al, 1994) Bax, (Krajewski et al, 1994), TGFβ, (Avery et al, 1993), and IGFBP-3 (Williams et al, 2000), have given important clues as to their function in the regulation of crypt architecture. Therefore we investigated the expression of pRb in the normal colonic crypt in vivo, and the mechanism of regulation of pRb expression with differentiation and apoptosis in vitro. Unlike previous reports (Ali et al, 1993; Kohn et al, 1997), we found that expression of the Rb protein in human colonic epithelial cells in vivo was not maximal in the differentiation zone, but that Rb protein was expressed throughout the base and mid crypt, and decreased significantly at the top of the normal crypt. Interestingly, during preparation of this manuscript, a study by Yamamoto et al (1999) was published in which down-regulation of Rb protein was also found at the top of the human colonic crypt. Using in vitro models to study the regulation of pRb expression in premalignant colonic epithelial cells, we found that the Rb protein was down-regulated in colonic adenoma-derived cell lines as differentiation was increased using both confluency and the differentiation agent sodium butyrate. However, interestingly, although differentiation could be induced in the tumorigenic cell line PC/AA/C1/SB10, the Rb protein was not significantly down-regulated. This finding suggests that suppression of pRb expression, although associated with, is not required for induction of differentiation and that regulation of the pRb expression levels is aberrant in tumorigenic cells. The aberrant expression in tumorigenic cells correlates with in vivo tumour tissue staining, where pRb is highly expressed throughout colorectal carcinomas. These results are contrary to findings in other in vitro cell systems such as normal adipose tissue, myocytes and haematopoietic cells, where differentiation is associated with the accumulation of *RB-1* mRNA (Coppola et al, 1990; Endo and Goto 1992; Richon et al, 1992; Slack et al, 1993; Chen et al, 1996), and where inactivation of the Rb protein is associated with tumorigenesis (Berns et al, 1995; Hiyana et al, 1995; Takano et al, 1995; Miyamoto et al, 1996). A major difference between the colon and other tissues is the notable lack of *RB-1* deletion or mutation in colorectal cancer (Meling et al, 1991; Ali et al, 1993) indicating that the inactivation of *RB-1* is not required for the development of colorectal tumorigenesis. In addition, the rise in the level of *RB-1* mRNA in colonic tumours compared to that in normal colonic mucosa (Gope et al, 1990), together with the associated increase in pRb phosphorylation (Gope and Gope, 1992) suggest that the active Rb protein is retained and indeed up-regulated in colonic tumorigenesis. This is further supported by the fact that Rb protein associated with elevated levels of transcripts appears to be normal size (4.7 kb) and functional (Ali et al, 1993), inferring that colorectal cancer cells retain functional pRb.

The role of Rb as a survival factor has been explored recently in more detail in a number of cell types; when the *RB-1* gene or Rb protein was absent from cells, or non-functional, apoptotic cell death increased (Almasan et al, 1995; Haas-Kogan et al, 1995), and mice deficient for *RB-1* are nonviable as a result of widespread apoptosis (Lee et al, 1992). In the current investigation we have shown that the human papilloma virus oncoprotein E7, which is known to bind and inactivate pRb, can increase both spontaneous and DNA damage-induced apoptosis in colonic adenoma-derived cells, suggesting that the suppression of the pRb expression in differentiated cells makes the cells more susceptible to apoptosis. Furthermore, cleaved products of the Rb protein have been detected in apoptotic cells harvested from our colonic cell lines (Browne et al, 1994), showing that cleavage of the Rb protein occurs during the apoptotic process. Browne et al (1994) suggested a role for pRb in controlling cell numbers in the colon, and hypothesize that pRb may act as a survival factor in the colon. Our evidence further suggests that pRb is acting as a survival factor in colonic epithelium; we propose that Rb protein

 *© 2001 Cancer Research Campaign*

expression is suppressed through a transcriptional mechanism as the cells near the top of the crypt, in order to 'prime' them for apoptotic cell death when they reach the gut lumen. Cells that retain pRb are protected from apoptosis because of the anti-apoptotic function of the Rb protein (Haas-Kogan et al, 1995). In support of this, Yamamota et al (1999) found that by targeting *RB-1* mRNA in HCT116 colon carcinoma cells with an anti-sense oligodeoxynucleotide, they could bring about a 70% decrease in the level of Rb protein expression which correlated with induction of apoptosis. This hypothesis provides an explanation for the high expression of functional Rb protein in colonic tumour cells, as retaining Rb protein would allow these cells to escape programmed cell death. Therefore our results, in pre-malignant cells using HPV-E7 to suppress pRb function, are in agreement with the findings of Yamamato et al (1999, published during preparation of this manuscript). This is of importance as both independent studies have shown that, contrary to the traditional role of pRb as a tumour suppressor, expression of the Rb protein may actually result in a growth advantage for colorectal cells.

In summary, Rb protein expression is down-regulated towards the top of the normal colonic crypt. Using in vitro models, we have shown that pRb expression in differentiated cells is transcriptionally regulated, and this level of regulation is reduced in tumorigenic cells. The functional consequence of pRb down-regulation is to increase the cellular response to apoptotic signals. Therefore down-regulation of pRb at the top of the colonic crypt may be important in 'priming' the cells for apoptosis at the lumenal surface, suggesting a role for pRb in the maintenance of normal tissue homeostasis of the colon. Expression of functional pRb in colorectal tumours, contrary to the traditional role of Rb as a tumour suppressor protein, would provide the cells with a survival advantage, allowing evasion of apoptotic signalling and hence leading to aberrant cell survival.

Finally, as suppression of pRb function by the HPV-E7 viral oncoprotein increased DNA damage-induced apoptosis, the expression of Rb protein by colorectal carcinoma cells may contribute to the resistance of tumours to current therapies. Hence, Rb protein may be a potentially important target for therapeutic intervention. As pRb acts as a survival factor in human colorectal tumour cells, then suppression of the Rb protein expression may increase the sensitivity of the cells to radiotherapy and/or chemotherapy, making treatment of colorectal cancer more effective.

### ACKNOWLEDGEMENTS

We would like to thank Maria Davies and Ian Paterson for their help with the Northern blotting analysis, and Jenny Baker and Ed Hayes for preparation of the immunohistochemical analysis. This work was supported by a program grant from the UK Cancer Research Campaign and by The Royal Society of Pathology and AstraZeneca Pharmaceuticals.

### REFERENCES

Ali AA, Marcus JN, Harvey JP, Roll R, Hodgson CP, Wildrick D, Chakraborty A and Boman BM (1993) RB1 protein in normal and malignant human colorectal tissue and colon cancer cell lines. *FASEB J* **7**: 931–937

Almasan A, Yin YX, Kelly RE, Lee EYHP, Bradley A, Li WW, Bertino JR and Wahl GM (1995) Deficiency of retinoblastoma protein leads to inappropriate S-phase entry, activation of E2F-responsive genes, and apoptosis. *Proc Natl Acad Sci USA* **92**: 5436–5440

An B and Dou QP (1996) Cleavage of retinoblastoma protein during apoptosis: interleukin-1 β-converting enzyme-like protease as candidate. *Cancer Res* **56**: 438–442

Avery A, Paraskeva C, Hall P, Flanders KC, Sporn M and Moorghen M (1993) TGF-β expression in the human colon – differential immunostaining along crypt epithelium. *Br J Cancer* **68**: 137–139

Beijersbergen RL and Bernards R (1996) Cell cycle regulation by the retinoblastoma family of growth inhibitory proteins. *Biochem Biophys Acta* **1287**: 103–120

Berns EMJJ, Deklein A, Vanputten WLJ, Vanstaveren IL, Bootsma A, Klijn JGM and Foekens JA (1995) Association between RB-1 gene alterations and factors of favourable prognosis in human breast cancer, without effect on survival. *Int J Cancer* **64**: 140–145

Bond JA, Haughton MF, Rowson JM, Smith PJ, Gire V, Wynford-Thomas D and Wyllie FS (1999) Control of replicative life span in human cells: Barriers to colonal expansion intermediate between M1 senescence and M2 crisis. *Mol Cell Biol* **19**: 3103–3114

Bracey TS, Williams AC and Paraskeva C (1997) Inhibition of radiation-induced G2 delay potentiates cell death by apoptosis and/or the induction of giant cells in colorectal tumour cells with disrupted p53 function. *Clin Can Res* **3**: 1371–1381

Browne SJ, Williams AC, Hague A, Butt AJ and Paraskeva C (1994) Loss of APC protein expressed by human colonic epithelial cells and the appearance of a specific low-molecular-weight form is associated with apoptosis *in vitro Int J Cancer* **59**: 56–64

Butt AJ (1996) The role of sodium butyrate and transforming growth factor β1 in growth control in colorectal carcinogenesis. PhD Thesis. University of Bristol, UK

Butt AJ, Hague A and Paraskeva C (1997) Butyrate- but not TGFβ1-induced apoptosis of colorectal adenoma cells is associated with increased expression of the differentiation markers E-cadherin and alkaline phosphatase. *Cell Death Different* **4**: 725–732

Chen P, Scully P, Shew J, Wang JYJ and Lee W (1989) Phosphorylation of the retinoblastoma gene product is modulated during cell cycle and cellular differentiation. *Cell* **58**: 1193–1198

Chen P, Riley DJ, Chen Y and Lee W (1996) Retinoblastoma protein positively regulates terminal adipocyte differentiation through direct interaction with C/EBPs. *Genes Dev* **10**: 2794–2804

Chen W-D, Otterson GA, Lipkowitz S, Khleif SN, Coxon AB and Kaye FJ (1997) Apoptosis is associated with cleavage of a 5 kDa fragment form RB which mimics dephosphorylation and modulates E2F binding. *Oncogene* **14**: 1243–1248

Clarke AR, Maandag ER, Vanroon M, Vanderlugt NMT, Vandervalk M, Hooper ML, Berns A and Riele HT (1992) Requirement for a functional Rb-1 gene in murine development. *Nature* **359**: 328–330

Cobrinik D, Lee M, Hannon G, Mulligan G, Bronson RT, Dyson N, Harlow E, Beach D, Weinberg RA and Jacks T (1996) Shared role of the pRB-related p130 and p107 proteins in limb development. *Genes Dev* **10**: 1633–1644

Coppola JA, Lewis BA, Cole MD, Ohnishi Y, Yamashita K and Monden Y (1990) Increased retinoblastoma gene expression is associated with late stages of differentiation in many different cell types. *Oncogene* **5**: 1731–1733

Cummings JH (1981) Short-chain fatty acids in the human colon. *Gut* **22**: 763–779

Dou QP and An B (1998) RB and apoptotic cell death. *Front Biosci* **3**: d419–d430

Endo T and Goto S (1992) Retinoblastoma gene product pRb accumulates during myogenic differentiation and is deinduced by the expression of SV40 large T-antigen. *J Biochem* **112**: 427–430

Field SJ, Tsai F-Y, Kuo F, Zubiaga AM, Kaelin WG, Livingston DM, Orkin SH and Greenberg ME (1996) E2F-1 functions in mice to promote apoptosis and suppress proliferation. *Cell* **85**: 549–561

Furukawa Y, DeCaprio JA, Freedman A, Kanakura Y, Nakamura M, Ernst TJ, Livingston DM and Griffin JD (1990) Expression and state of phosphorylation of the retinoblastoma suscptibility gene product in cycling and noncycling human hematopoietic cells. *Proc Natl Acad Sci USA* **87**: 2770–2774

Gaverelli Y, Sherman Y and Ben-Sasson SA (1992) Identification of programmed cell death in situ via specific labeling of nuclear DNA fragmentation. *J Cell Biol* **119**: 493–501

Gope R and Gope ML (1992) Abundance and state of phosphorylation of the retinoblastoma susceptibility gene product in human colon cancer. *Mol and Cell Biochem* **110**: 123–133

Gope R, Christensen MA, Thorson A, Lynch HT, Smyrk T, Hodgson C, Wildrick DM, Gope ML and Boman BM (1990) Increased expression of the retinoblastoma gene in human colorectal carcinomas relative to normal colonic mucosa. *J Natl Cancer Inst* **82**: 310–314

© 2001 Cancer Research Campaign 

Gu W, Schneider JW, Condorelli G, Kaushal S, Mahdavi V and Nadal Ginard B (1993) Interaction of myogenic factors and the retinoblastoma protein mediates muscle cell commitment and differentiation. *Cell* **72**: 309–324

Haas-Kogan DA, Kogan SC, Levi D, Dazin P, Tang A, Fung YKT and Israel MA (1995) Inhibition of apoptosis by the retinoblastoma gene product. *EMBO J* **14**: 461–472

Hague A, Manning AM, Hanlon KA, Huschtscha LI, Hart D and Paraskeva C (1993) Sodium butyrate induces apoptosis in human colonic tumour cell lines in a p53-independent pathway – implications for the possible role of dietary fibre in the prevention of large-bowel cancer. *Int J Cancer* **55**: 498–505

Hague A, Moorghen M, Hicks D, Chapman M and Paraskeva C (1994) BCL-2 expression in human colorectal adenomas and carcinomas. *Oncogene* **9**: 3367–3370

Hall PA, Coates PJ, Ansari B and Hopwood DJ (1994) Regulation of cell number in the mammalian gastrointestinal tract: The importance of apoptosis. *J Cell Science* **107**: 3569–3577

Hiyana K, Ishioka S, Shirotani Y, Inai K, Hiyama E, Murakami I, Isobe T, Inamizu T and Yamakido M (1995) Alterations in telomeric repeat length in lung cancer are associated with loss of heterozygosity in p53 and Rb. *Oncogene* **10**: 937–944

Jacks T, Fazeli A, Schmitt EM, Bronson RT, Goodell MA and Weinberg RA (1992) Effects of an Rb mutation in the Mouse. *Nature* **359**: 295–300

Kalman D, Whittaker K, Bishop JM and O'Lague PH (1993) Domains of E1A that bind p105Rb, p130, and p300 are required to block nerve growth factor-induced neurite growth in PC12 cells. *Mol Biol Cell* **4**: 353–361

Kirkland S (1985) Dome formation by a human colonic adenocarcinoma cell line (HCA-7). *Cancer Res* **45**: 3790–3795

Kohn GJ, Schwartz HJ, Ruebner BH, Wong AJ and Lawson MJ (1997) Colonic retinoblastoma protein and proliferation in cancer and non-cancer patients. *J Gastroenterol Hepatol* **12**: 198–203

Krajewski S, Krajewska M, Shabaik A, Miyashita T, Wang HG and Reed JC (1994) Immunohistochemical determination of *in vivo* distribution of Bax, a dominant inhibitor of Bcl-2. *Am J Pathol* **145**: 1323–1336

Leder A and Leder P (1975) Butyric Acid, a potent inducer of erythroid differentiation in cultured erythroleukemic cells. *Cell* **5**: 319–322

Lee EYHP, Chang CY, Hu NP, Wang YCJ, Lai CC, Herrup K, Lee WH and Bradley A (1992) Mice deficient for Rb are nonviable and show defects in neurogenesis and haematopoiesis. *Nature* **359**: 288–294

Li H, Kawasaki H, Nishida E, Hattori S and Nakamura S (1996) Ras-regulated hypophosphorylation of the retinoblastoma protein mediates neuronal differentiation in PC12 cells. *Neurochemistry* **66**: 2287–2294

Meling GI, Lothe RA, Borresen AL, Hauge S, Graue C, Clausen OPF and Rognum TO (1991) Genetic alterations within the retinoblastoma locus in colorectal carcinomas – relation to DNA ploidy pattern studied by flow cytometric analysis. *Br J Cancer* **64**: 475–480

Merritt AJ, Potten CS, Kemp CJ, Hickman JA, Balmain A, Lane DP, and Hall PA (1994) The role of p53 in spontaneous and radiation-induced apoptosis in the gastrointestinal tract of normal and p53-deficient mice. *Cancer Research* **54**: 614–617

Miyamoto H, Shuin T, Ikeda I, Hosaka M and Kubato Y (1996) Loss of heterozygosity at the p53, Rb, DCC and APC tumour-suppressor gene loci in human bladder-cancer. *J Urology* **155**: 1444–1447

Mulligan G and Jacks T (1998) The retinoblastoma gene family: cousins with overlapping interests. *Trends in Genetics* **14**: 223–229

Paggi MG, Baldi A, Bonetto F and Giordano A (1996) Retinoblastoma protein family in cell cycle and cancer: A Review. *J Cell Biochemistry* **62**: 418–430

Paraskeva C, Buckle BG, Sheer D and Wigley CB (1984) The isolation and characterization of colorectal epithelial cell lines at different stages in malignant transformation from familial polyposis coli patients. *Int J Cancer* **34**: 49–56

Prasad K and Sinha PK (1976) Effect of sodium butyrate on mammalian cells in culture: a review. *In Vitro* **12**: 125–132

Qin X, Livingston DM, Kaelin WG and Adams PD (1994) Deregulated transcription factor E2F-1 expression leads to S-phase entry and p53-mediated apoptosis. *Proc Natl Acad Sci USA* **91**: 10918–10922

Richon VM, Rifkind RA and Marks PA (1992) Expression and phosphorylation of the retinoblastoma protein during induced differentiation of murine erythroleukaemia cells. *Cell Growth Differ* **3**: 413–420

Riley DJ, Liu CY and Lee WH (1997) Mutations of N-terminal regions render the retinoblastoma protein insufficient for functions in development and tumor suppression. *Mol Cell Biol* **17**: 7342–7352

Sellers WR and Kaelin WG (1996) RB as a modulator of transcription. *Biochim Biophys Acta* **1288**: M1–M5

Sellers WR, Notvitch BG, Miyake S, Heith A, Otterson GA, Kaye FJ, Lassar AB and Kaelin WGJ (1998) Stable binding to E2F is not required for the retinoblastoma protein to activate transcription, promote differentiation, and suppress tumor cell growth. *Genes and Dev* **12**: 95–106

Slack RS, Hamel PA, Bladon TS, Gill RM and McBurney MW (1993) Regulated expression of the retinoblastoma gene in differentiating embryonic carcinoma cells. *Oncogene* **8**: 1585–1591

Takano H, Okamoto A, Terashima Y and Yokota J (1995) High-incidence of allelic loss at the RB gene locus in advanced human ovarian cancer. *Int J Oncology* **6**: 129–135

Tsujii and Dubois (1995) Alterations in cellular adhesion and apoptosis in epithelial cells overexpressing prostaglandin endoperoxide synthase 2. *Cell* **83**: 493–501

Weinberg RA (1995) The retinoblastoma protein and cell cycle control. *Cell* **81**: 323–330

Wildrick DM and Boman BM (1994) Does the human retinoblastoma gene have a role in colon cancer? *Mol Carcinogen* **10**: 1–7

Williams AC, Harper SJ and Paraskeva C (1990) Neoplastic transformation of a human colonic epithelial cell line: *in vitro* evidence for the adenoma to carcinoma sequence. *Cancer Res* **50**: 4724–4730

Williams AC, Collard TJ and Paraskeva C (1999) An acidic environment leads to p53 dependent induction of apoptosis in human adenoma and carcinoma cell lines: Implications for clonal selection during colorectal carcinogenesis. *Oncogene* **18**: 3199–3204

Williams AC, Collard TJ, Perks CM, Newcombe P, Moorghen M, Holly JMP and Paraskeva C (2000) Increased p53 dependent apoptosis by the insulin-like growth factor binding protein IGFBP-3 in human colonic adenoma derived cells. *Cancer Res* **60**: 22–27

Yamamoto H, Monden T, Ikeda K, Izawa H, Fukuda K, Fukunaga M, Tomita N, Shimano T, Shiozaki H and Monden M (1995) Coexpression of cdk2/cdc2 and retinoblastoma gene products in colorectal cancer. *Br J Cancer* **71**: 1231–1236

Yamamoto H, Soh J, Monden T, Klein MG, Zhang LM, Shirin H, Arber N, Tomita N, Schieren I, Stein CA and Weinstein IB (1999) Paradoxical increase in retinoblastoma protein in colorectal carcinomas may protect cells from apoptosis. *Clinical Cancer Res* **5**: 1805–1815

Yee AS, Shih HH and Tevosian SG (1998) New perspectives on retinoblastoma family functions in differentiation. *Frontiers in Bioscience* **3**: 532–547

Zackenhaus E, Jiang Z, Chung D, Marth JD, Phillips RA and Gallie BL (1996) pRb controls proliferation, differentiation and death of skeletal muscle cells and other lineages during embryogenesis. *Genes Dev* **10**: 3051–3064

*© 2001 Cancer Research Campaign*
